# Pheochromocytoma presenting as recurrent urinary tract infections : a case report

**DOI:** 10.1186/1752-1947-5-6

**Published:** 2011-01-12

**Authors:** Roisin T Dolan, Joseph S Butler, Gerard P McEntee, Maria M Byrne

**Affiliations:** 1 Department of Surgery, Mater Misericordiae University Hospital, Eccles Street Dublin 7, Ireland; 2 Department of Endocrinology, Mater Misericordiae University Hospital, Eccles Street, Dublin 7, Ireland

## Abstract

**Introduction:**

Pheochromocytomas are rare, potentially fatal, neuroendocrine tumors of the adrenal medulla or extra-adrenal paraganglia. Their clinical presentation varies greatly from the classic triad of episodic headache, diaphoresis and tachycardia to include a spectrum of non-specific symptomatology.

**Case presentation:**

A 43-year-old Caucasian woman was referred to us from primary care services with a three-month history of recurrent urinary tract infections on a background of hypertension, latent autoimmune diabetes of adulthood and autoimmune hypothyroidism. At 38 years she required insulin therapy. Despite medication compliance and dietary control, she reported a recent history of increased insulin requirements and uncontrolled hypertension with concomitant recurrent urinary tract infections. A renal ultrasound examination, to rule out underlying renal pathology, revealed an incidental 8cm right adrenal mass of both solid and cystic components. A subsequent computed tomography of her abdomen and pelvis confirmed a solid heterogeneous mass consistent with a pheochromocytoma. There were no other features suggestive of multiple endocrine neoplasia. Urinary collection over 24 hours revealed grossly elevated levels of catecholamines and metabolites. Following an open right adrenalectomy, our patient's insulin requirements were significantly reduced and her symptoms resolved. Two weeks post-operatively, an iodine-131-metaiodobenzylguanidine scintigraphy was negative for residual tumor and metastatic disease. Urinary catecholamine and metabolite concentrations were within the normal range at a follow-up six months later.

**Conclusion:**

Pheochromocytoma is a rare catecholamine-producing tumor requiring a high index of suspicion for early diagnosis. Our case report serves to highlight the importance of considering pheochromocytoma as a differential diagnosis in the atypical setting of recurrent urinary tract infections and concomitant autoimmune disease.

## Introduction

Pheochromocytoma is a rare, insidious adrenal medullary neuroendocrine tumor representing approximately 5% of adrenal incidentalomas [1]. It is a sympathetic paraganglioma of chromaffin cell origin and catecholamine hypersecretion is a common clinical manifestation [2,3]. Today, 25% of all pheochromocytomas are discovered incidentally during imaging studies for unrelated disorders.

Clinical awareness of the variable manifestations of this insidious tumor is key for early diagnosis and optimal patient management. Clinical presentation varies from the classic triad of episodic headache, diaphoresis and tachycardia to include a spectrum of non-specific symptomatology. Despite improved diagnostic techniques, there remains an approximate delay of three years between initial symptoms and a final diagnosis.

We discuss an atypical case of a 43-year-old woman presenting with recurrent urinary tract infections in the setting of an undiagnosed pheochromocytoma. We review the contribution of catecholamine hypersecretion to patient symptomatology and the unique association of pheochromocytoma with other autoimmune endocrinopathies.

## Case Presentation

A 43-year-old Caucasian woman presented to our primary care services with a three-month history of recurrent, laboratory confirmed *Escherichia coli *urinary tract infections. She was diagnosed with gestational diabetes at age 33 years, and in the postpartum period, diet controlled type 2 diabetes mellitus and concomitant hypertension were confirmed. She required insulin therapy some five years later, and, following seropositivity to anti-glutamic acid decarboxylase antibodies, was diagnosed with latent autoimmune diabetes of adulthood (LADA). In addition, she developed autoimmune hypothyroidism at age 38 years. Despite medication compliance and dietary control, she reported a recent history of increased insulin requirements and uncontrolled hypertension with concomitant recurrent urinary tract infections. Our patient is not sexually active.

**Figure 1:**
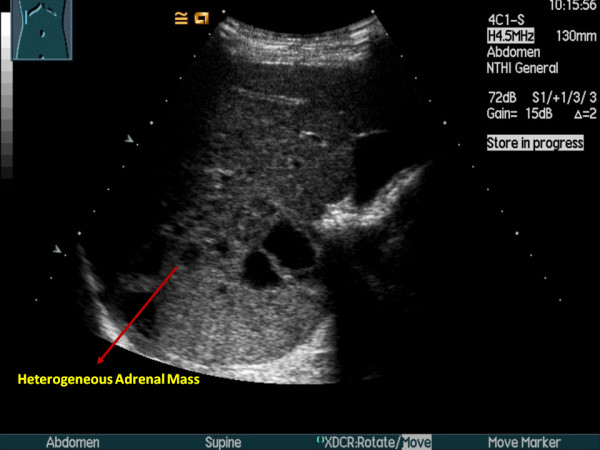
Abdominal ultrasound depicting a large mixed cystic/solid right adrenal mass

**Figure 2:**
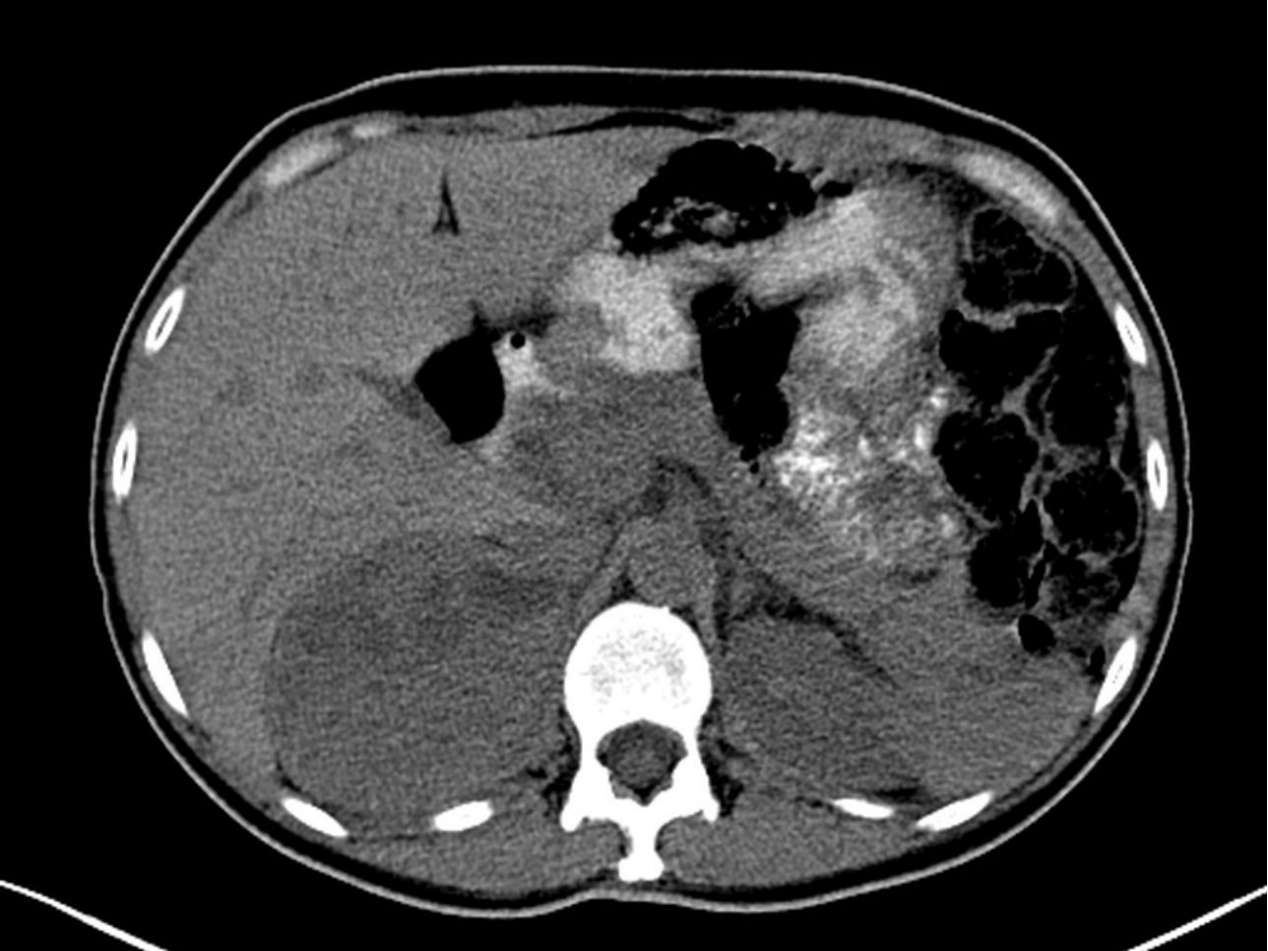
**CT abdomen further illustrating a solid heterogeneous right adrenal mass with focal cystic degeneration, slightly displacing the right kidney**.

**Figure 3:**
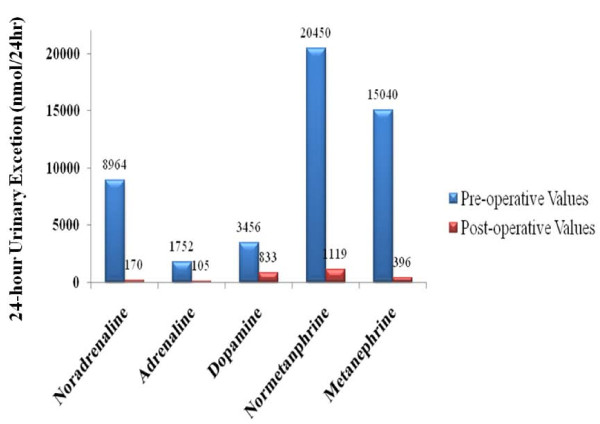
**Catecholamine and metabolite concentrations before and after adrenalectomy, taken from 24-hour urine sample**. Normal ranges: noradrenaline (0-900nmol/24h); adrenaline (0-230nmol/24h); dopamine (0-3300 nmol/24h); normetanephrine (50-2800nmol/24h); metanephrine (25-1800nmol/24h).

Renal ultrasound examination, to rule out underlying renal pathology, revealed an incidental 8cm right adrenal mass of both solid and cystic components (Figure 1). A subsequent non-contrast computed tomography (CT) of the abdomen and pelvis confirmed a solid heterogeneous mass consistent with a pheochromocytoma, 9cm in maximal dimension. No regional adenopathy, vascular invasion or metastatic disease was evident (Figure 2). There were no other features suggestive of multiple endocrine neoplasia. On physical examination, her blood pressure was elevated at 160/90mmHg with no orthostatic changes. Her fasting blood glucose was 12mmol/L and her glycated hemoglobin level (HbA1C) was 9.9%. Her body mass index was within the normal range at 22.5. A 24-hour urinary collection provided biochemical confirmation with grossly elevated levels of catecholamines and metabolites (Figure 3).

**Figure 4:**
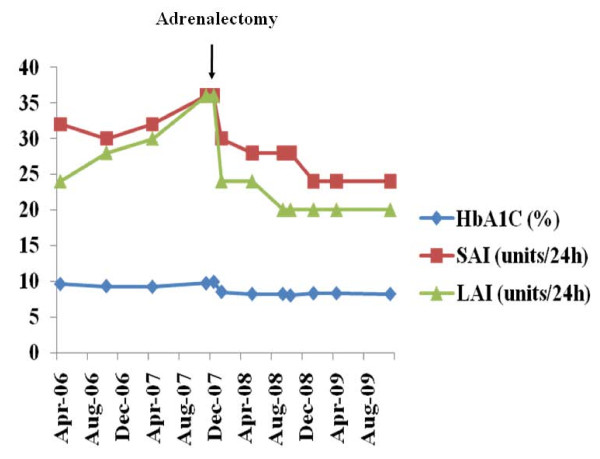
**Graph displaying the dramatic reduction in insulin requirements and HbA1C following adrenalectomy for pheochromocytoma**.

Following a 10-day course of phenoxybenzamine α-adrenergic blockade, the patient underwent an open right adrenalectomy. The immediate post-operative period was complicated by profound episodes of hypotension, requiring three days of inotropic support in high dependency care. Her recovery after this period was uncomplicated with an observed reduction in insulin requirements and anti-hypertensive therapy (Figure 4).

**Figure 5:**
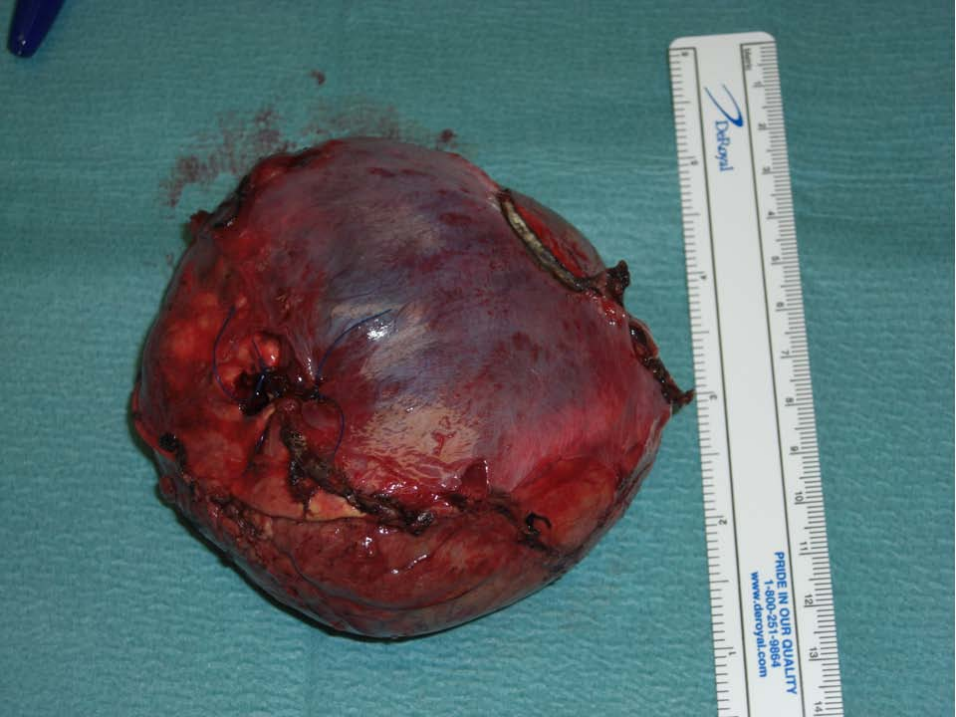
**Gross adrenal pheochromocytoma specimen following surgical excision**.

**Figure 6:**
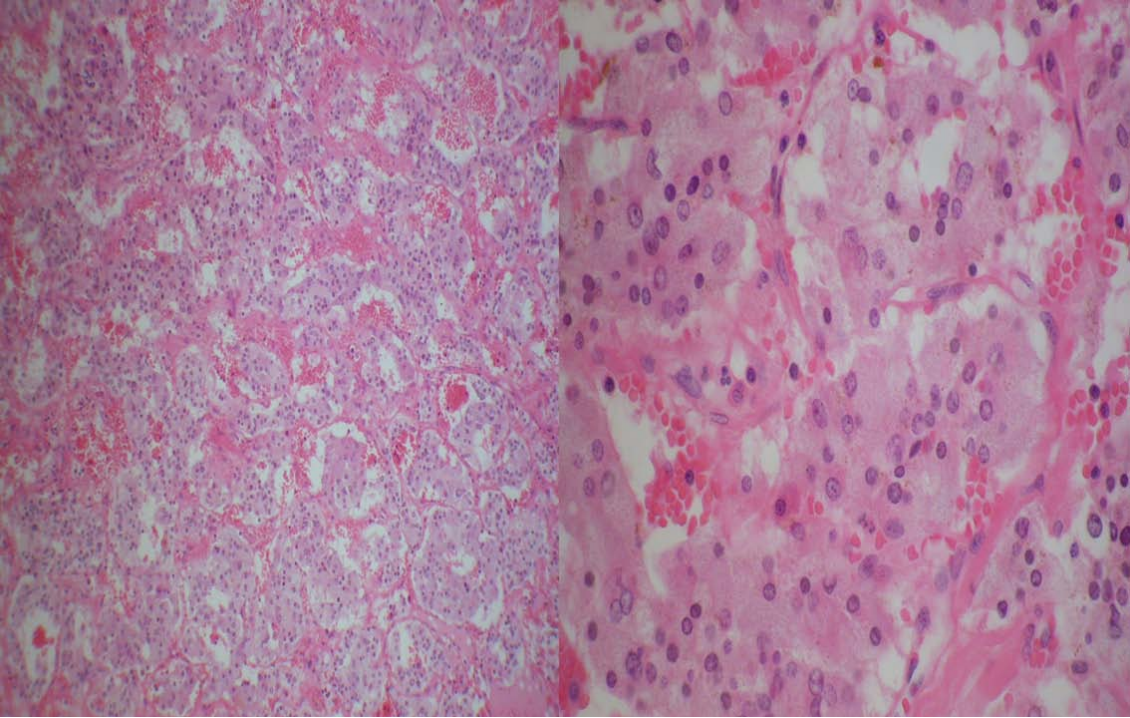
**(a) Low power (10X) image depicting nests of tumour cells with prominent intervening capillary network giving characteristic Zellballen appearance; (b) High power (20X) image highlighting the presence of abundant finely granular amphophilic cytoplasm, round nuclei, marked focal nuclear pleomorphism and tumor giant cells.** These findings do not correlate with malignant behavior.

**Figure 7:**
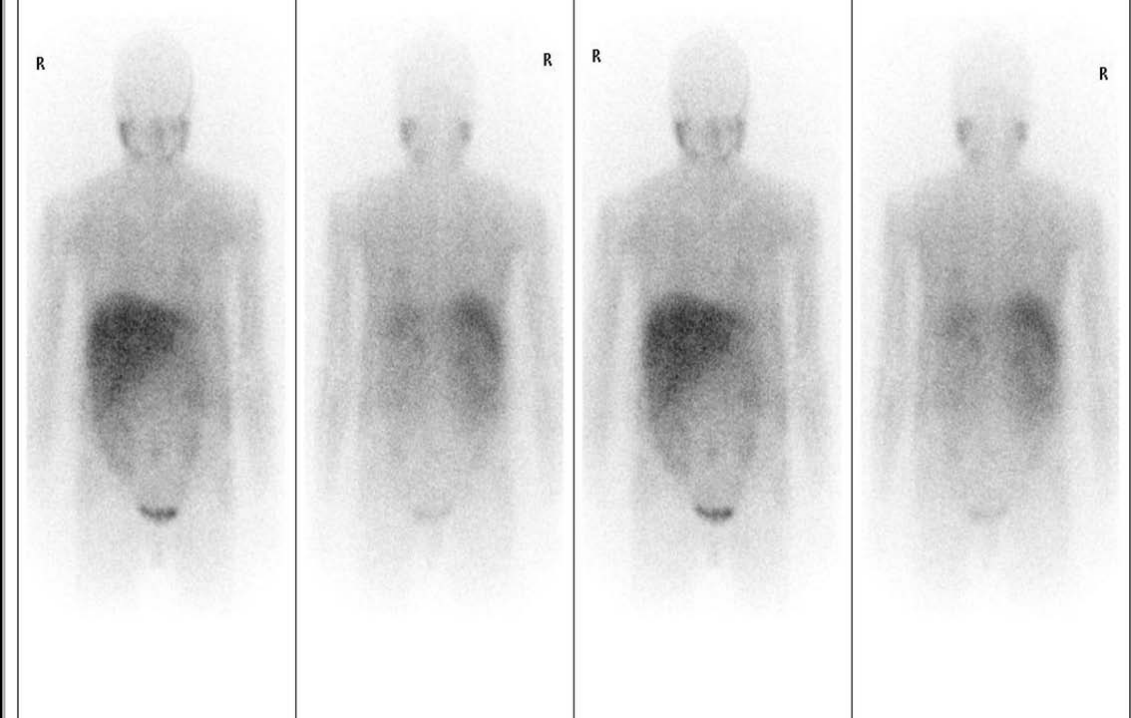
**MIBG scintigraphy two weeks post-adrenalectomy displaying no evidence of residual chromaffin cell uptake or metastatic disease**.

Histopathological examination of the excised tissue confirmed a 9.5x9.6cm solid heterogeneous tumor arising from her right adrenal medulla (Figure 5). Microscopic evaluation revealed Zellballen cellular architecture, a distinct characteristic of paragangliomas. There was no invasion of adjacent tissues or blood vessels and the MIB-1 proliferation index was 3%, favoring benign behavior (Figure 6). Iodine-131-metaiodobenzylguanidine (MIBG) scintigraphy performed two weeks post-operatively confirmed complete excision with no evidence of metastatic disease (Figure 7). Two years post-operatively our patient is symptom-free with normal urinary catecholamines and metabolites. She denies any further recurrence of urinary tract infections post-operatively. She continues to take anti-hypertensive and insulin therapy.

## Discussion

Pheochromocytomas are rare, adrenal medullary neuroendocrine tumors with a worldwide incidence of two to eight per million which peaks in the fourth and fifth decades of life [4,5]. Forty percent of pheochromocytomas are asymptomatic and discovered incidentally during radiologic imaging [4,5]. Forty percent of pheochromocytomas present with the classic triad of paroxysmal headache, diaphoresis and tachycardia. Hypertensive crisis may develop in some patients resulting in cardiovascular shock with stroke, myocardial infarction, or multiple organ failure [5,6]. Pheochromocytoma detection poses an immense clinical challenge as these insidious neoplasms can masquerade as a spectrum of non-specific symptomatologies; numerous atypical presentations of this unique neoplasm have been reported in the literature [6-11].

The lethal nature of this neoplasm is dependent on two major characteristics. The first of these is its ability to secrete excess catecholamines, resulting in potentially catastrophic consequences. The second is its malignant potential. In this context, a multidisciplinary team approach involving anesthesia, surgical and endocrinological expertise is necessary to maximize optimal patient outcome [3,12]. Surgical resection is the definitive treatment for patients with pheochromocytoma [3,12]. With increasing worldwide expertise, laparoscopic adrenalectomy is now regarded as the gold standard for approximately 60% of patients requiring adrenalectomy including pheochromocytoma [13]. It is recommended that only experienced surgeons perform the procedure and that the approach take into account the type, size, site and hereditary background of the tumor [12]. However, open trans-peritoneal surgery is indicated when tumors are multiple, large tumors of a size greater than 8 to 10cm, or when the tumor will be difficult to remove using laparoscopy [12,13]. In our case, an open approach was undertaken to reduce intra-operative mechanical mobilization of the large tumor, which can potentially stimulate surges of catecholamine secretion, precipitating hypertensive crises and fatal arrhythmias. It is recommended that all patients receive 7 to 10 days of α-adenoreceptor antagonists pre-operatively to prevent catecholamine-induced vasoconstriction. There is no specific recommendation on the preferred drugs for pre-operative blockade; α-adenoreceptor antagonists, calcium-channel blockers and angiotension-receptor blockers have all been proven beneficial [12].

The association of pheochromocytoma with other endocrinopathies is a rare yet recognized phenomenon. Previous studies have concluded that diabetes mellitus is present in up to a third of patients presenting with pheochromocyotma [7,8,14]. This umbrella term encompasses type 1, type 2 and gestational diabetes mellitus. Catecholamine overproduction leads to both decreased insulin secretion and increased peripheral resistance to insulin action, by stimulating α2 and β2 adenoreceptors respectively. Following adrenalectomy, some patients revert to normoglycamia with a normal glucose tolerance test, yet a proportion of patients continue to display features of glucose intolerance, although to a lesser extent, as demonstrated in our case [7,8]. It has been reported that a proportion of this latter group display seropositivity to pancreatic islet cell auto-antibodies [8]. Recent literature has also reported a rare association of autoimmune hypo- and hyperthyroidism with pheochromocytoma [9]. Thus, a high index of suspicion for pheochromocytoma should be maintained in the context of concomitant endocrinopathies and typical or atypical symptomatology.

To the best of our knowledge, there have been no previous cases reported in the current literature in which a patient presents with recurrent urinary tract infections in association with pheochromocytoma. We postulate that catecholamine overproduction in pheochromocytoma causes decreased insulin secretion and increased end-organ resistance, resulting in hyperglycaemia. Studies have demonstrated impairment of host defenses, including decreased polymorphonuclear leukocyte mobilization, chemotaxis, and phagocytic activity related to hyperglycemia [15]. Recent literature has reported persistent neutrophilia as a preceding symptom of pheochromocytoma. However, catecholamine overproduction is known to provoke neutrophilia and mimic sepsis, thus a laboratory confirmed urinary tract infection with an identified causative agent is necessary in the context of this unusual tumor.

## Conclusion

Pheochromocytoma is a rare catecholamine-producing tumor with potential life-threatening consequences. Clinical manifestations unique to this tumor are occasional atypical and non-specific symptomatology and its association with autoimmune disorders. A multidisciplinary approach involving anesthesia, endocrinology and surgical expertise is the gold standard in maximizing patient care. This case exhibits a rare combination of pheochromocytoma with LADA and autoimmune hypothyroidism. Here we highlight the importance of considering pheochromocytoma as a differential diagnosis in the setting of recurrent urinary tract infections and concomitant autoimmune endocrinopathies.

## Consent

Written informed consent was obtained from the patient for publication of this case report and any accompanying images. A copy of the written consent is available for review by the Editor-in-Chief of this journal.

## Competing interests

The authors declare that they have no competing interests.

## Authors' contributions

RD conceived the study, acquired patient data and drafted the manuscript. JB critically reviewed the manuscript. All authors contributed intellectual content and have read and approved the final manuscript.
